# Supplementary material for: Structural Insights into the Protein Mannosyltransferase from Mycobacterium tuberculosis reveal a WW-Domain-Like Protein Motif in Bacteria
Source: Commun Biol. 2025 Aug 7;8:1175. doi: 10.1038/s42003-025-08593-9 (PMC12331936; doi:10.1038/s42003-025-08593-9)
Supplement: Supplementary file 2 — Supplemental Material [file 42003_2025_8593_MOESM2_ESM.pdf]

# Fig S1

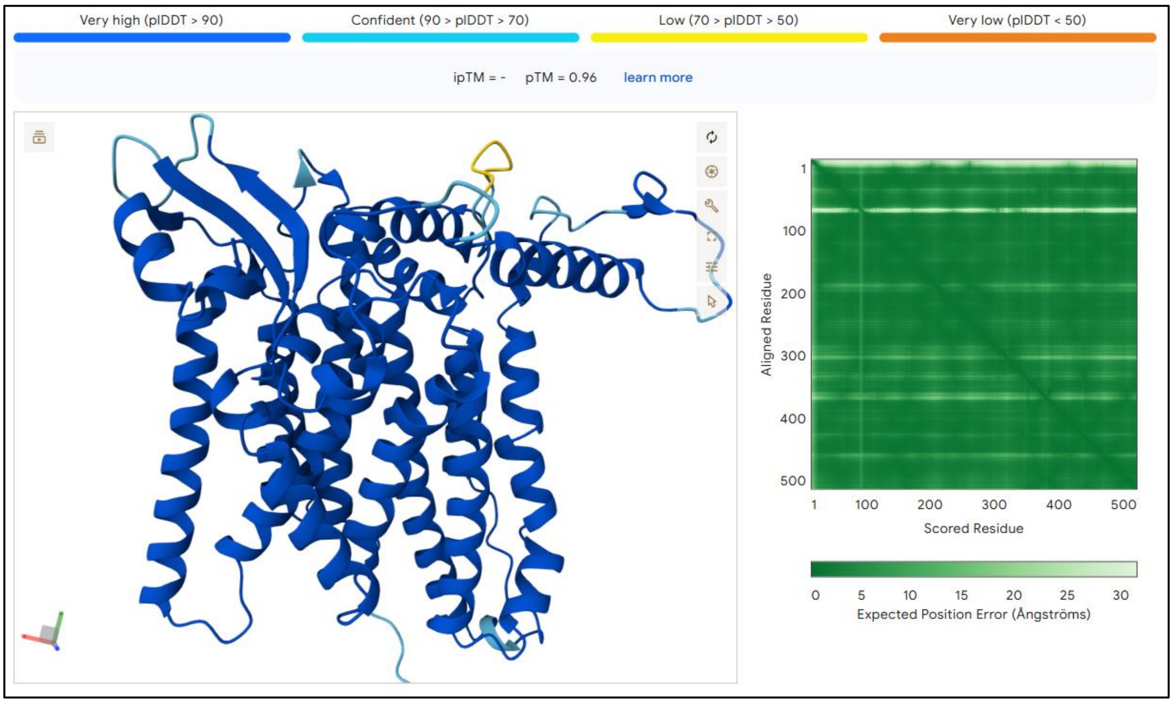


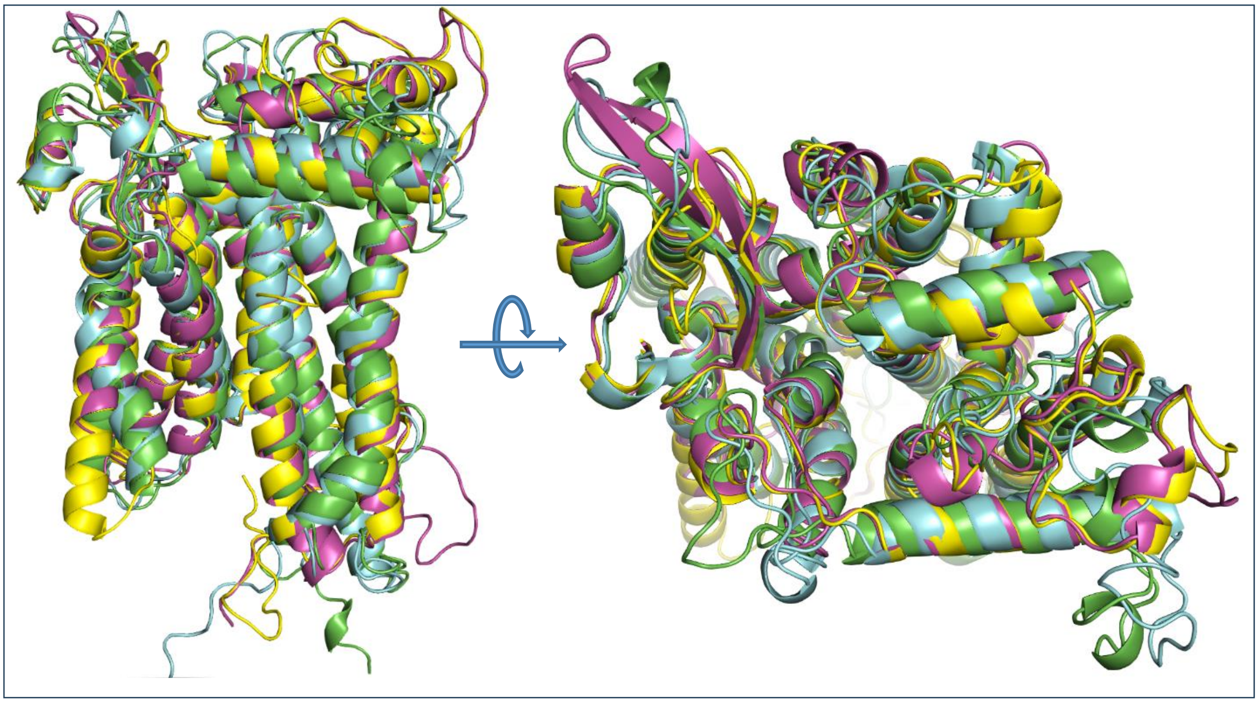

| Model 1 | Model 2 | RMSD | Atoms | Cycles |
|---|---|---|---|---|
| AlphaFold3(Cyan) | Phyre2(Pink) | 1.966 | 2,611 | 7 |
| AlphaFold3 (Cyan) | I-TASSER(Yellow) | 1.987 | 2,126 | 13 |
| AlphaFold3 (Cyan) | Robeta(Green) | 1.51 | 3,176 | 5 |
| Robeta(Green) | I-TASSER(Yellow) | 2.278 | 3,596 | 13 |
| Robeta(Green) | Phyre2(Pink) | 2.025 | 2,577 | 8 |
| I-TASSER(Yellow) | Phyre2(Pink) | 1.098 | 2,456 | 5 |

**Figure S1:**: screenshot of the AlphaFold 3 MtPMT model figuring the model quality metrics; down; pairwise comparisons of the different models generated using the Phyre2 [33], Robetta [34], I-TASSER [35] and AlphaFold3 web-based protein 3D structure prediction softwares showing the degres of structural similarity calculated (Graphics generated with PyMOL Molecular Graphics System, Version 2.5.8 Schrödinger, LLC).

# Fig S2

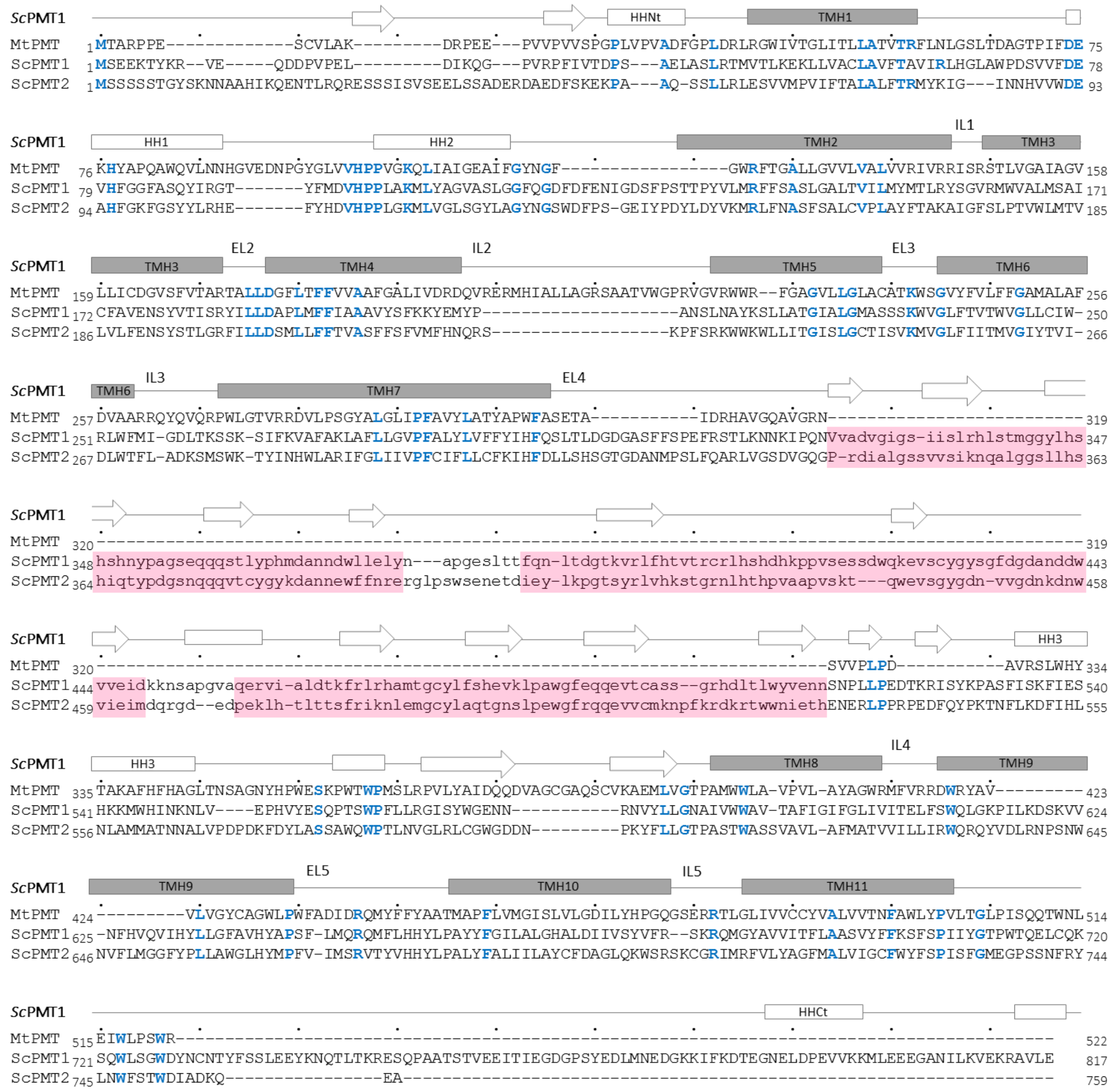


**Figure S2:** Sequence alignments of bacterial MtPMT and yeast ScPMT1-2. Secondary structures of ScPMT1 are annotated; the rectangles represent α helices and the arrows β strands (EL: external loop; HH: horizontal helix; IL: internal loop; TMH: transmembrane helix; Nt: N terminus; Ct: C terminus). Amino acids highlighted in blue are strictly conserved. MIR domains are annotated by lowercase letters, highlighted in pink and are defined as delimited in the “ProRule” database.

Fig S3

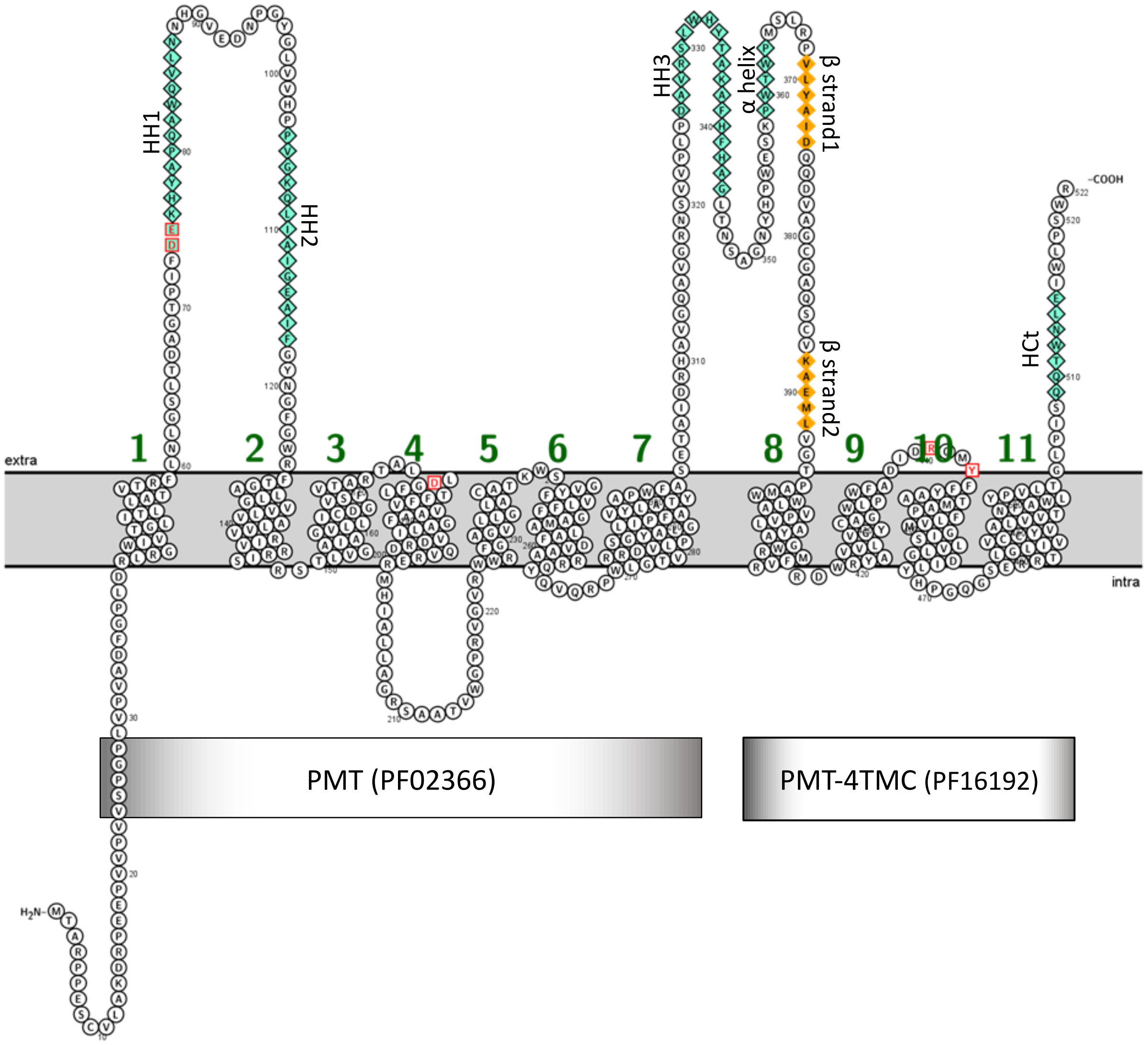


**Figure S3:** MtPMT “Protter” topology diagram highlighting in red the conserved amino-acids presumed to be involved in the mannose transfer reaction and outlining the sequence of the secondary structures and of the eleven TMHs delimited according the AlphaFold model AF-P9WN05-F1 (the successive TMHs correspond to the amino acids sequences 40-59, 126-147, 150-171, 175-200, 223-238, 242-264, 270-302, 396-416, 419-437, 445-468 and 474-503).

Fig S4

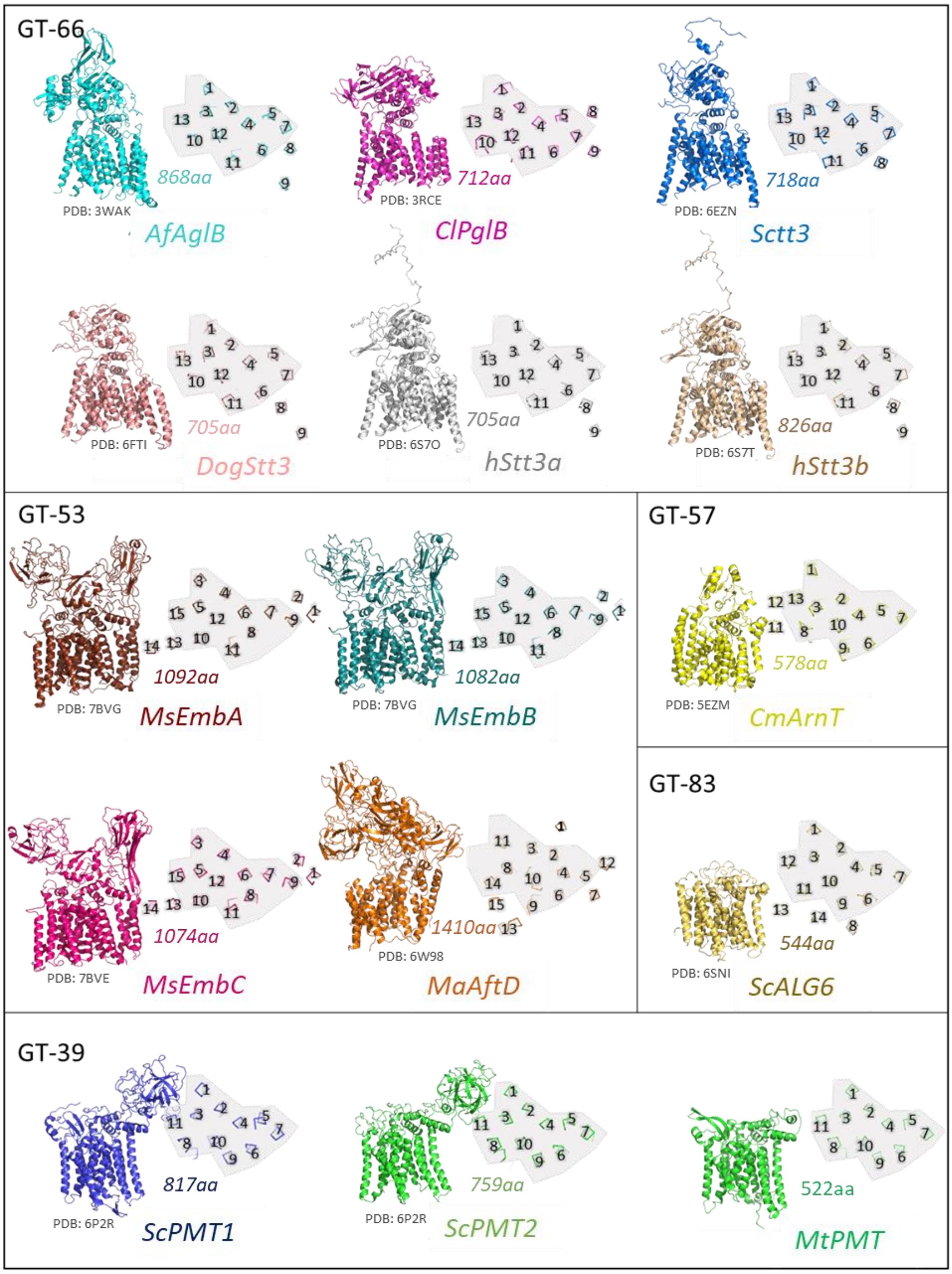


**Figure S4: Resolved structure and topologies of members from different CAZY GT clades of the GT-C family highlighting the elementary character of the bacterial MtPMT fold.** The structures are presented as ribbon diagrams (left) and as slices through the transmembrane helices, seen from above (right) (Sc: *S. cerevisiae*; Ms: *M. smegmatis*; Ma: *M. abscessus*; Af: *A. fulgidus*; Cl: *C. lari*; h: *human*; Cm: *C. metalidurans*). The conserved GT-C module of 11 transmembrane domains is shaded. Transmembrane helices are numbered. PDB IDs are indicated as well as the number of amino acids (aa).

Fig S5

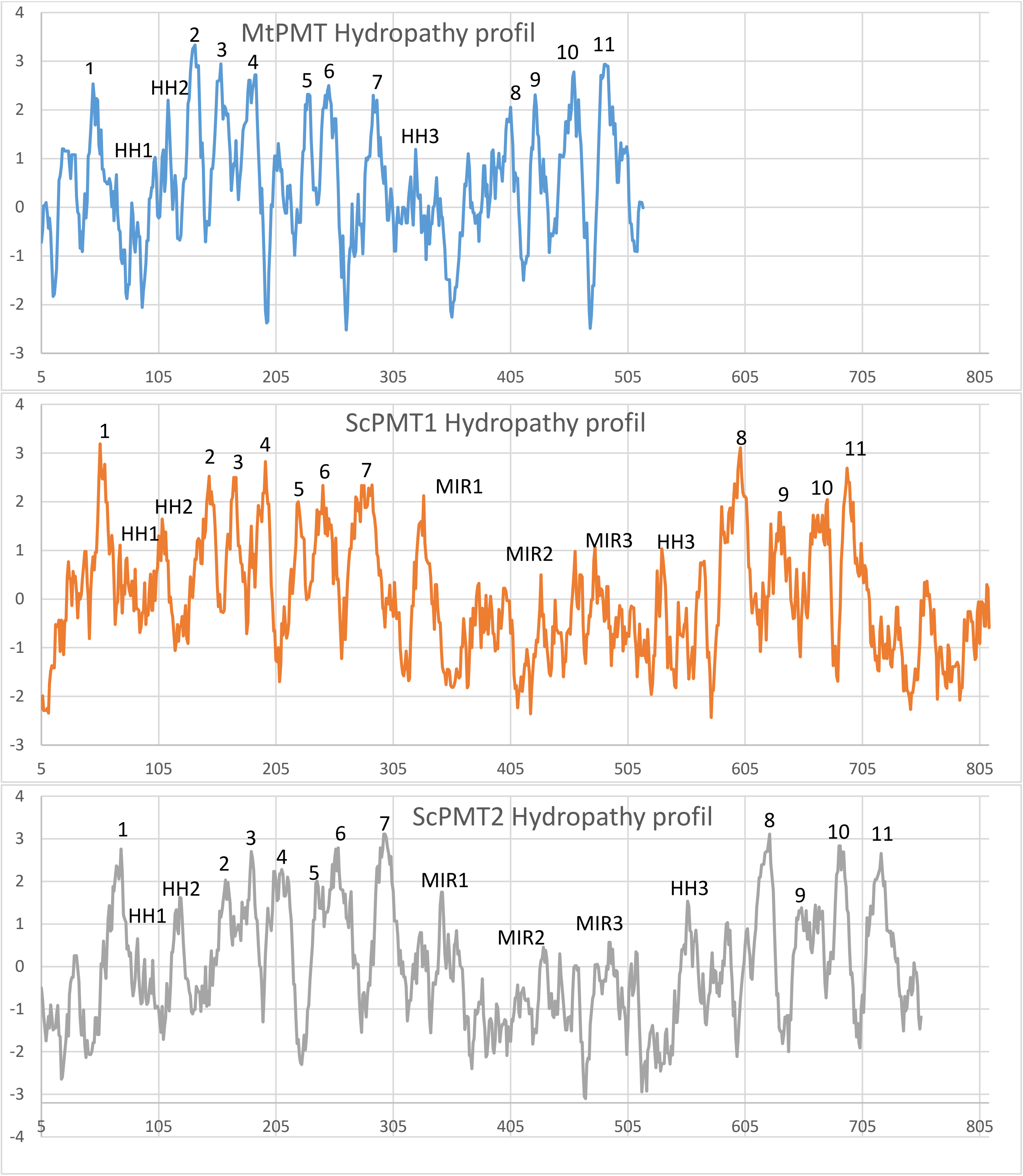


**Figure S5:** Comparison of the sequence derived hydropathy profiles of the bacterial MtPMT to the yeast ScPMTs evidencing the MtPMT lower polar balance resulting from the absence of the large soluble MIR domains (indicated numbers corresponds to the TMHs numbering; graph generated with the GRAVY calculator https://www.bioinformatics.org/sms2/protein_gravy.html.)

Fig S6

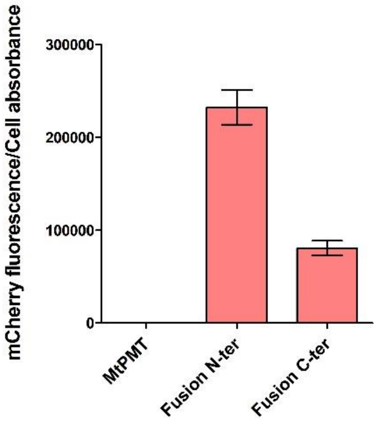


**Figure S6**: Normalized fluorescence of *M. smegmatis* ΔMsPMT cells ectopically expressing the mCherry-$^{Nter}$MtPMT or MtPMT$^{Cter}$-mCherry fusion proteins showing a significant quantitative difference consistent with an alternate subcellular location of the fluorescent protein in the cytoplasm or in the periplasm.

## Fig S7

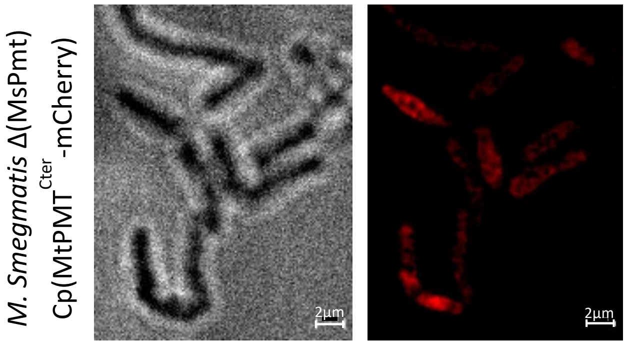


**Figure S7**: Transmission and fluorescence microscopy *M. smegmatis* ΔMsPmt bacteria expressing the MtPMT$^{Cter}$-mCherry fusion protein. Cell showing low fluorescence insufficient for high resolution imaging processing (Scale bars : 1 µm).

# Fig S8

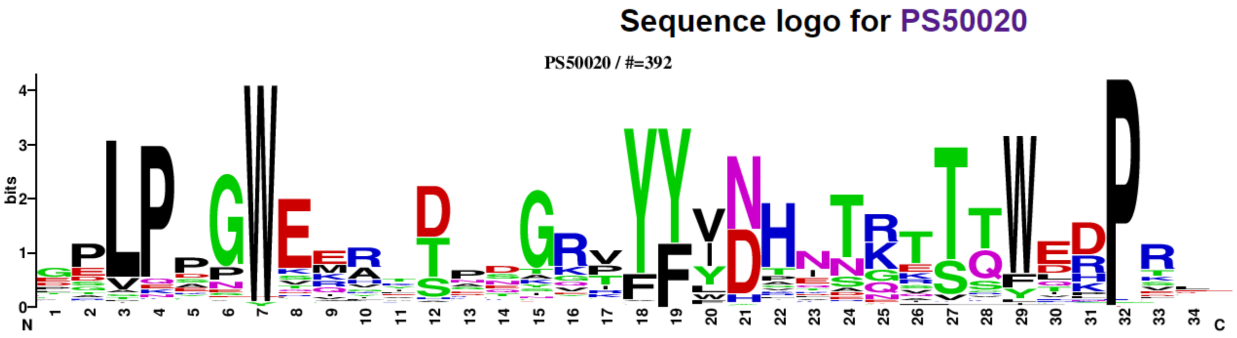


**Figure S8**: Consensus sequence of the ProSite's PS50020 module deduced from the alignment of 392 primary WW motif sequences detected by automatic annotation from 227 eukariotic true protein sequences. (Sequence Logo established from ProSite database)

## Fig S9

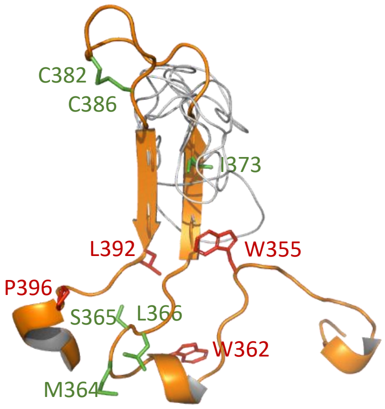


**Figure S9**: Close-up view of the AlphaFold model of the MtPMT EL4Cter domain as a ribbon diagram showing the location of mycobacteria-specific amino acids (green) and conserved amino acids essential for MtPMT activity (red) tested herein. The grey lines correspond to different conformations of the EL4Cter hairpin segment between I373 and M391 of the MtPMT AlphaFold model, pointing out the relative flexibility of this domain.

Fig S10

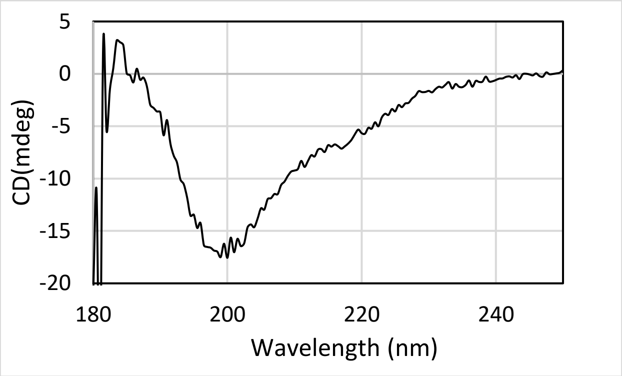


**Figure S10:** Circular dichroism spectrum of peptide EL4Cter* (0.4mg/mL in H2O) obtained with a Jasco spectrometer. The results are expressed by the average ellipticity of the residues [Θ] (in deg.cm2/dmol) as a function of the wavelength.

23

Fig S11

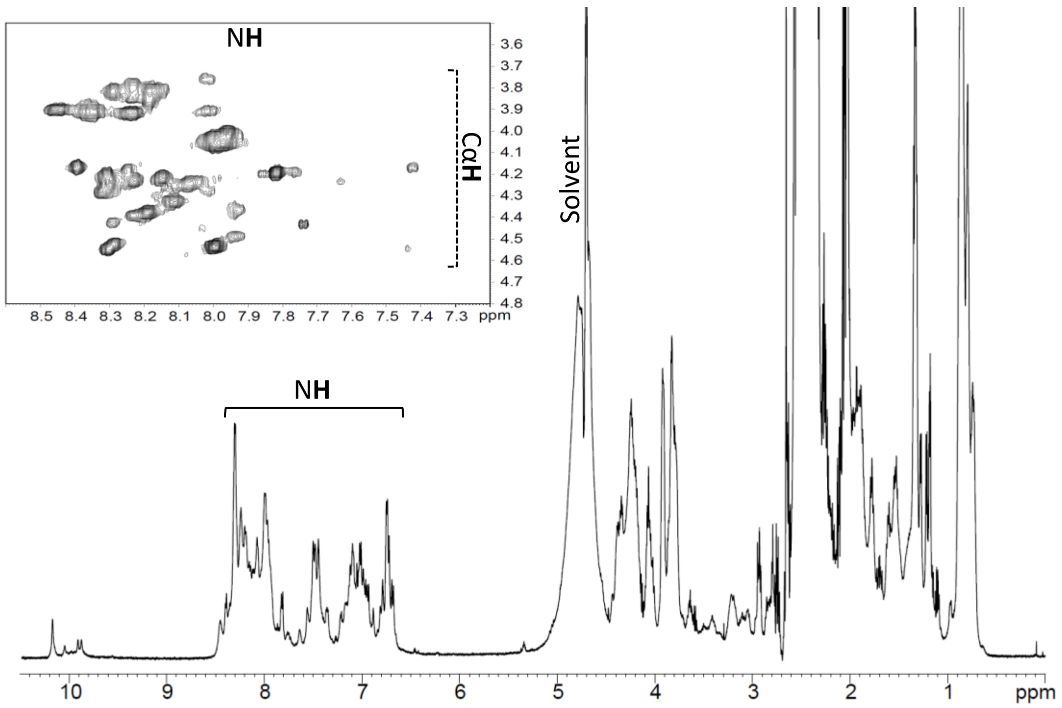


**Figure S11:** 1H NMR analysis of the peptide EL4Cter*. 1D $^{1}$H NMR spectrum 600MHz (3.2mg/ml, 20mM Phosphate Buffer in 90/10 $H_2O/D_2O$, pH6.5, 300K). Inset: partial 2D NMR spectrum $^{1}$H-$^{1}$H TOCSY 80ms, corresponding to the NH-CαH correlation zone (top panel).

Fig S12

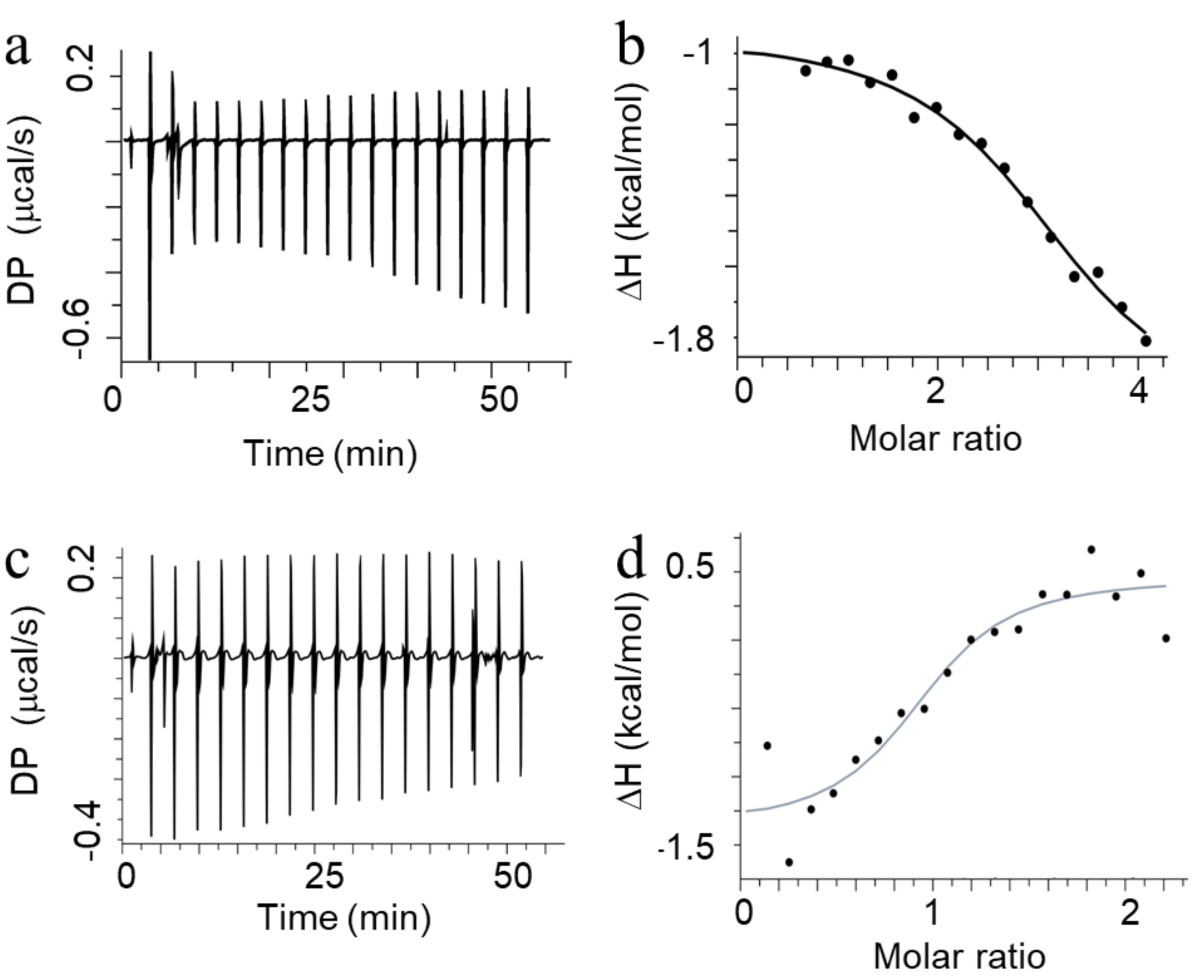


**Figure S12:** Isothermal titration calorimetry (ITC) analysis of the interaction at 37°C between the Apa1 ligand and the synthetic EL4Cter* peptide (a, b; ratio peptide/ligand: 33/700), or its W10A mutant analogue (W10A)EL4Cter* (c, d; ratio peptide/ligand: 25/288)

**Table S1:** Entire protein high-resolution MS relative abundances of the purified FasC$^{His}$ glycoforms produced by the *M. smegmatis* devoid of PMT (ΔPMT) or expressing the heterologous MtPMT, mCherry-MtPMT and MtPMT-PhoA. Values are the mean ± sd of the relative abundance measured on 3 independent MS Spectra.

| | Mass (Da) | MtPMT WT Mean relative Intensity | MtPMT-PhoA Mean relative Intensity | mCherry-MtPMT Mean relative Intensity | ΔMtMT Relative Intensity |
|---|---|---|---|---|---|
| ○ | 23,840 | 10.153 +/- 0.6 | 13.337 +/- 0.3 | 13.060 +/- 2.0 | 100 |
| ▽ | 24,010 | 5.533 +/- 0.2 | 7.225 +/- 0.2 | 4.293 +/- 0.4 | |
| △ | 24,170 | 6.435 +/- 0.3 | 9.513 +/- 0.3 | 4.94 +/- 0.1 | |
| ▷ | 24,330 | 9.853 +/- 0.4 | 13.15 +/- 0.3 | 8.39 +/- 0.3 | |
| □ | 24,490 | 8.803 +/- 0.1 | 10.543 +/- 0.2 | 7.77 +/- 0.3 | |
| ◊ | 24,660 | 7.808 +/- 0.1 | 8.258+/- 0.1 | 7.728 +/- 0.1 | |
| ☆ | 24,820 | 7.105 +/- 0.1 | 7.575 +/- 0.1 | 7.258 +/- 0.1 | |
| ○ | 24,980 | 6.758 +/- 0.2 | 6.56 +/- 0.1 | 7.25 +/- 0.1 | |
| ▽ | 25,140 | 5.413 +/- 0.1 | 4.915 +/- 0.2 | 6.185 +/- 0.1 | |
| △ | 25,300 | 4.715 +/- 0.2 | 3.81 +/- 0.1 | 5.443 +/- 0.1 | |
| ▷ | 25,470 | 4.46 +/- 0.1 | 3.225 +/- 0.1 | 5.298 +/- 0.2 | |
| □ | 25,630 | 4.975 +/- 0.2 | 3.35 +/- 0.2 | 5.69 +/- 0.2 | |
| ◊ | 25,790 | 3.638 +/- 0.2 | 2.02 +/- 0.1 | 2.870 +/- 0.2 | |
| ☆ | 25,950 | 4.255 +/- 0.1 | 2.308 +/- 0.1 | 4.58 +/- 0.2 | |
| ○ | 26,120 | 1.963 +/- 0.1 | 0.803 +/- 0.1 | 2.443 +/- 0.2 | |
| ▽ | 26,280 | 2.538 +/- 0.1 | 0.91 +/- 0.1 | 3.015 +/- 0.2 | |
| △ | 26,440 | 2.025 +/- 0.1 | 0.665 +/- 0.1 | 2.285 +/- 0.1 | |
| ▷ | 26,600 | 0.810 +/- 0.09 | | 1.375 +/- 0.09 | |
| □ | 26,760 | 1.030 +/- 0.06 | | 0.948 +/- 0.06 | |

**Table S2**: Names and sequences of primers used in this study. Primers used for directed mutagenesis of the vector encoding MtPMT have underlined sequences that correspond to mutations, and sequence highlighted in gray indicates restriction sites added or removed through silent mutation for controlling the insertion of the mutation by restriction profile.

| Name | Sequence 5’-3’ | Feature |
|---|---|---|
| Directed mutagenesis | | |
| D74N | CC GAT GCC GGG ACC CCC ATC TTC AAC GAG AAG CAT TAC G | Remove NaeI Site |
| D74E | CC GAT GCC GGG ACC CCC ATC TTC GAG GAG AAG CAT TAC | Remove NaeI Site |
| D74A | CC GAT GCC GGG ACC CCC ATC TTC GCT GAG AAG CAT TAC | Remove NaeI Site |
| DE74-75ED | CC GAT GCC GGG ACC CCC ATC TTC GAG GAC AAG CAT TAC | Remove NaeI Site |
| D176N | G ACC GCG CTG TTA AAC GGC TTC CTG | Remove MseI Site |
| D176E | CC GCG CTG CTC GAG GGC TTC CTG | Add XhoI site |
| D176A | CC GCG CTG CTG GCC GGC TTC CTG | Add NaeI site |
| R441L | GAC ATC GAC CTG CAG ATG TAC | Add PstI site |
| R441K | CC GAC ATC GAT GCG CAG ATG TAC | Add ClaI site |
| R441A | CC GAC ATC GAT AAG CAG ATG TAC | Add ClaI site |
| Y444A | GCC GAC ATC GAT CGG CAG ATG GCC TTC TTC TAC | Add PvuI site |
| Y444F | GCC GAC ATC GAT CGG CAG ATG TTC TTC TTC TAC | Add PvuI site |
| W355A | GGC AAC TAC CAC CCT GCA GAA TCC AAA CCG TGG | Add PstI site |
| W355F | GGC AAC TAC CAT CCT TTC GAA TCC AAA CCG | Add FokI site |
| P359A | TCC AAA GCG TGG ACC TGG CCC ATG TCG TTG CGC CCG GTG | Add Eco52I site |
| W362A | CCG TGG ACC GCG CCC ATG TCG TTG CGG CCG GTG CTC TAC GCC ATC | Add SduI site |
| W362F | CCG TGG ACC TTT CCC ATG TCG TTG CGG CCG GTG CTC TAC GCC ATC | Add SduI site |
| M364A | CC TGG CCC GCG TCG TTG CGG CCG GTG CTC TAC GCC ATC G | Add SduI site |
| M364V | CC TGG CCC GTG TCG TTG CGG CCG GTG CTC TAC GCC ATC G | Add SduI site |
| S365A | CC TGG CCC ATG GCG TTG CGG CCG GTG CTC TAC GCC ATC G | Add SduI site |
| S365T | CC TGG CCC ATG ACG TTG CGG CCG GTG CTC TAC GCC ATC G | Add SduI site |
| L366A | GG CCC ATG TCG GCG CGG CCG GTG CTC TAC GCC ATC G | Add SduI site |
| Y371A | G TTG CGG CCG GTG CTC GCC GCC ATC GAC CAG | Add SduI site |
| Y371F | G TTG CGG CCG GTG CTC TTC GCC ATC GAC CAG C | Add SduI site |
| I373Q | G CCG GTG CTC TAC GCC CAG GAC CAG CAA G | Add SduI site |
| I373L | G CCG GTG CTC TAC GCC CTC GAC CAG CAA G | Add SduI site |
| C381A | GTT GCA GGC GCC GGT GCA CAG TCG TGC | Add ApaLI site |
| C381S | GTT GCA GGC AGC GGT GCA CAG TCG TGC | Add ApaLI site |
| C386A | GC TGC GGT GCA CAG TCG GCC GTC AAG G | Add ApaLI site |
| C386S | GC TGC GGT GCA CAG TCG TCC GTC AAG G | Add ApaLI site |
| L392A | AAG GCC GAG ATG GCG GTG GGC ACG CCC GCG ATG | Add SduI site |
| L392W | AAG GCC GAG ATG TGG GTG GGC ACG CCC GCG ATG | Add SduI site |
| P396A | GAG ATG CTG GTG GGC ACG GCC GCG ATG TGG | Add BseSI site |
| Fusion protein | | |
| Rev_mC_C_plasmid | CCT TGC TCA CGG ATC CGC TGC TGC CCC AGC TGG GCA GCC AGA TC | Add mcherry at the Cterminus of MtPMT |
| For_mC_C_plasmid | TGA AGC TTA TCG ATG TCG ACG TAG | |
| Rev_mC_C_insert | GTC GAC ATC GAT AAG CTT CAC TTG TAC AGC TCG TCC ATG | |
| For_mC_C_insert | GCC CAG CTG GGG CAG CAG CGG ATC CGT GAG CAA GGG CGA GGA G | |
| Rev_mC_N_plasmid | CAT ATG TAC TTC TCC TTC TAA TCC ATT GG | Add mcherry at the Nterminus of MtPMT |
| For_mC_N_plasmid | GCT GTA CAA GGG CAG CAG CGG ATC CGT ACC CGT CGT CAG CCC C | |
| Rev_mC_N_insert | CGA CGG GTA CGG ATC CGC TGC TGC CCT TGT ACA GCT CGT CCA TG | |
| For_mC_N_insert | TAG AAG GAG AAG TAC ATA TGG TGA GCA AGG GCG AGG AG | |
| Rev_PhoA_C_plasmid | CCA GCT GGG CAG CCA GAT C | Add PhoA at the Cterminus of MtPMT |
| For_PhoA_C _plasmid | AAG CCG CTC TGG GGC TGA AAT GAA GCT TAT CGA TGT CGA CGT AG | |
| Rev_PhoA_C _insert | TTT CAG CCC CAG AGC GGC | |
| For_PhoA_C _insert | AGA TCT GGC TGC CCA GCT GGG ACT CTT ATA CAC AAG TAG CGT CC | |
| Rev_PhoA_N _plasmid | CGC TAC TTG TGT ATA AGA GTC CAT ATG TAC TTC TCC TTC TAA | Add PhoA at the Nterminus of MtPMT |
| For_PhoA_N _plasmid | AAA GCC GCT CTG GGG CTG AAA GTA CCC GTC GTC AGC CCC | |
| Rev_PhoA_N _insert | CGC TAC TTG TGT ATA AGA GTC CAT ATG TAC TTC TCC TTC TAA | |
| For_PhoA_N _insert | AAA GCC GCT CTG GGG CTG AAA GTA CCC GTC GTC AGC CCC | |

**Table S3**: Names and main features of plasmids used in this study

| Name | Main features | Source |
|---|---|---|
| **pWM158** | Mycobacterial integrative plasmid, derived from pMV361, containing the wild-type MtPMT gene from Mtb (Rv1002c) under the control of the pBlaF* promotor | Liu *et al*. 2013 |
| **pMV361** | Integrative Escherichia coli/mycobacteria shuttle vector | Stover *et al*. 1991 |
| **pWM218** | Mycobacterial replicative plasmid containing the Msmeg FasCHis gene under the control of the pBlaF* promotor | Liu *et al*. 2013 |
| **pWM158**-D74N | Derived from pWM158, containing the D74N-mutated-MtPMT gene from Mtb | This study |
| **pWM158**-D74E | Derived from pWM158, containing the D74E-mutated-MtPMT gene from Mtb | This study |
| **pWM158**-D74A | Derived from pWM158, containing the D74A-mutated-MtPMT gene from Mtb | This study |
| **pWM158**-DE74-75ED | Derived from pWM158, containing the DE74-75ED-mutated-MtPMT gene from Mtb | This study |
| **pWM158**-D176N | Derived from pWM158, containing the D176N-mutated-MtPMT gene from Mtb | This study |
| **pWM158**-D176E | Derived from pWM158, containing the D176E-mutated-MtPMT gene from Mtb | This study |
| **pWM158**-D176A | Derived from pWM158, containing the D176A-mutated-MtPMT gene from Mtb | This study |
| **pWM158**-R441L | Derived from pWM158, containing the R441L-mutated-MtPMT gene from Mtb | This study |
| **pWM158**-R441K | Derived from pWM158, containing the R441K-mutated-MtPMT gene from Mtb | This study |
| **pWM158**-R441A | Derived from pWM158, containing the R441A-mutated-MtPMT gene from Mtb | This study |
| **pWM158**-Y444A | Derived from pWM158, containing the Y444A-mutated-MtPMT gene from Mtb | Geraud *et al*. 2023 |
| **pWM158**-Y444F | Derived from pWM158, containing the Y444F-mutated-MtPMT gene from Mtb | This study |
| **pWM158**-W355A | Derived from pWM158, containing the W355A-mutated-MtPMT gene from Mtb | This study |
| **pWM158**-W355F | Derived from pWM158, containing the W355F-mutated-MtPMT gene from Mtb | This study |
| **pWM158**-P359A | Derived from pWM158, containing the P359A-mutated-MtPMT gene from Mtb | This study |
| **pWM158**-W362A | Derived from pWM158, containing the W362A-mutated-MtPMT gene from Mtb | This study |
| **pWM158**-W362F | Derived from pWM158, containing the W362F-mutated-MtPMT gene from Mtb | This study |
| **pWM158**-M364A | Derived from pWM158, containing the M364A-mutated-MtPMT gene from Mtb | This study |
| **pWM158**-M364V | Derived from pWM158, containing the M364V-mutated-MtPMT gene from Mtb | This study |
| **pWM158**-S365A | Derived from pWM158, containing the S365A-mutated-MtPMT gene from Mtb | This study |
| **pWM158**-S365T | Derived from pWM158, containing the S365T-mutated-MtPMT gene from Mtb | This study |
| **pWM158**-L366A | Derived from pWM158, containing the L366A-mutated-MtPMT gene from Mtb | Geraud *et al*. 2023 |
| **pWM158**-Y371A | Derived from pWM158, containing the Y371A-mutated-MtPMT gene from Mtb | Geraud *et al*. 2023 |
| **pWM158**-Y371F | Derived from pWM158, containing the Y371F-mutated-MtPMT gene from Mtb | This study |
| **pWM158**-I373Q | Derived from pWM158, containing the I373Q-mutated-MtPMT gene from Mtb | This study |
| **pWM158**-I373L | Derived from pWM158, containing the I373L-mutated-MtPMT gene from Mtb | This study |
| **pWM158**-C381A | Derived from pWM158, containing the C381A-mutated-MtPMT gene from Mtb | This study |
| **pWM158**-C381S | Derived from pWM158, containing the C381S-mutated-MtPMT gene from Mtb | This study |
| **pWM158**-C386A | Derived from pWM158, containing the C386A-mutated-MtPMT gene from Mtb | This study |
| **pWM158**-C386S | Derived from pWM158, containing the C386S-mutated-MtPMT gene from Mtb | This study |
| **pWM158**-C381-386A | Derived from pWM158, containing the C381-386A-mutated-MtPMT gene from Mtb | This study |
| **pWM158**-C381-386S | Derived from pWM158, containing the C381-386S-mutated-MtPMT gene from Mtb | This study |
| **pWM158**-L392A | Derived from pWM158, containing the L392A-mutated-MtPMT gene from Mtb | This study |
| **pWM158**-L392W | Derived from pWM158, containing the L392W-mutated-MtPMT gene from Mtb | This study |
| **pWM158**-Y371A-L392A | Derived from pWM158, containing the Y371A-L392A-mutated-MtPMT gene from Mtb | This study |
| **pWM158**-P396A | Derived from pWM158, containing the P396A-mutated-MtPMT gene from Mtb | This study |
| **pWM158-N-mCherry** | Derived from pWM158, with mCherry at the N-terminal extremity of MtPMT | This study |
| **pWM158-C-mCherry** | Derived from pWM158, with mCherry at the C-terminal extremity of MtPMT | This study |
| **pWM158-N-PhoA** | Derived from pWM158, with PhoA at the N-terminal extremity of MtPMT | This study |
| **pWM158-C-PhoA** | Derived from pWM158, with PhoA at the C-terminal extremity of MtPMT | This study |
| **pWM158**-D74N-**PhoA** | Derived from pWM158-C-PhoA, containing the D74N-mutated-MtPMT gene from Mtb | This study |
| **pWM158**-D176N-**PhoA** | Derived from pWM158-C-PhoA, containing the D176N-mutated-MtPMT gene from Mtb | This study |
| **pWM158**-R441L-**PhoA** | Derived from pWM158-C-PhoA, containing the R441L-mutated-MtPMT gene from Mtb | This study |
| **pWM158**-Y444A-**PhoA** | Derived from pWM158-C-PhoA, containing the Y444A-mutated-MtPMT gene from Mtb | This study |
| **pWM158**-W355A-**PhoA** | Derived from pWM158-C-PhoA, containing the W355A-mutated-MtPMT gene from Mtb | This study |
| **pWM158**-W355F-**PhoA** | Derived from pWM158-C-PhoA, containing the W355F-mutated-MtPMT gene from Mtb | This study |
| **pWM158**-P359A-**PhoA** | Derived from pWM158-C-PhoA, containing the P359A-mutated-MtPMT gene from Mtb | This study |
| **pWM158**-W362A-**PhoA** | Derived from pWM158-C-PhoA, containing the W362A -mutated-MtPMT gene from | This study |
| **pWM158**-W362F-**PhoA** | Derived from pWM158-C-PhoA, containing the W362F -mutated-MtPMT gene from Mtb | This study |
| **pWM158**-Y371A-**PhoA** | Derived from pWM158-C-PhoA, containing the Y371A -mutated-MtPMT gene from Mtb | This study |
| **pWM158**-Y371F-**PhoA** | Derived from pWM158-C-PhoA, containing the Y371F -mutated-MtPMT gene from Mtb | This study |
| **pWM158**-L392A-**PhoA** | Derived from pWM158-C-PhoA, containing the L392A -mutated-MtPMT gene from Mtb | This study |
| **pWM158**-L392W-**PhoA** | Derived from pWM158-C-PhoA, containing the L392W -mutated-MtPMT gene from Mtb | This study |
| **pWM158**-Y371A-L392A-**PhoA** | Derived from pWM158-C-PhoA, containing the Y371A-L392A-mutated-MtPMT gene from Mtb | This study |
| **pWM158**-P396A-**PhoA** | Derived from pWM158-C-PhoA, containing the P396A -mutated-MtPMT gene from Mtb | This study |
| **pMmpS4-PhoA** | Derived from pWM158, expressing MmpS4 with PhoA at its C-terminal extremity | This study |
| **pKatG-PhoA** | Derived from pWM158, expressing KatG with PhoA at its C-terminal extremity | This study |
| **pMIPS4PhoA** | Derived from pMIP12e, expressing KatG1 with PhoA at its C-terminal extremity | [39] |
| **pMIPKatGPhoA** | Derived from pMIP12e, expressing Mmps4 with PhoA at its C-terminal extremity | [39] |

References

[1] Liu CF, Tonini L, Malaga W, Beau M, Stella A, Bouyssie D, et al. Bacterial protein-O-mannosylating enzyme is crucial for virulence of Mycobacterium tuberculosis. Proc Natl Acad Sci U S A. 2013;110:6560-5.
[2] Bai L, Kovach A, You Q, Kenny A, Li H. Structure of the eukaryotic protein O-mannosyltransferase Pmt1-Pmt2 complex. Nat Struct Mol Biol. 2019;26:704-11.
[3] Smith GT, Sweredoski MJ, Hess S. O-linked glycosylation sites profiling in Mycobacterium tuberculosis culture filtrate proteins. J Proteomics. 2014;97:296-306.
[4] Tonini L, Sadet B, Stella A, Bouyssie D, Nigou J, Burlet-Schiltz O, Riviere M. Potential Plasticity of the Mannoprotein Repertoire Associated to Mycobacterium tuberculosis Virulence Unveiled by Mass Spectrometry-Based Glycoproteomics. Molecules. 2020;25.
[5] Madeira F, Madhusoodanan N, Lee J, Eusebi A, Niewielska A, Tivey ARN, et al. The EMBL-EBI Job Dispatcher sequence analysis tools framework in 2024. Nucleic Acids Res. 2024;52:W521-W5.
