# Supplementary material for: Structural Insights into the Protein Mannosyltransferase from Mycobacterium tuberculosis reveal a WW-Domain-Like Protein Motif in Bacteria
Source: Commun Biol. 2025 Aug 7;8:1175. doi: 10.1038/s42003-025-08593-9 (PMC12331936; doi:10.1038/s42003-025-08593-9)
Supplement: Supplementary file 3 — Description of Additional Supplementary Materials [file 42003_2025_8593_MOESM3_ESM.pdf]

Description of Additional Supplementary Files

File name: Supplementary Data 1

Description: numerical data for graphs Fig2c; Fig3a,b,d; Fig 5d,e;

File name: Supplementary Data 2

Description: Table of peptide sequences underlying Fig4a

File name: Supplementary Data 3

Description: numerical data from Fig6a to e

File name: Supplementary Data 4

Description: Table of peptide sequences underlying Fig7

File name: Supplementary Data 5

Description: numerical data from Fig S12a

File name: Supplementary Data 6

Description: numerical data from Fig S12b
