# Supplementary material for: Structural Insights into the Protein Mannosyltransferase from Mycobacterium tuberculosis reveal a WW-Domain-Like Protein Motif in Bacteria
Source: Commun Biol. 2025 Aug 7;8:1175. doi: 10.1038/s42003-025-08593-9 (PMC12331936; doi:10.1038/s42003-025-08593-9)
Supplement: Supplementary file 4 — Supplementary Data 1 [file 42003_2025_8593_MOESM4_ESM.pdf]

Table data for graph figure 2c : Biological replicate values of the PhoA activity quantified in the perplasmic fractions of *M. smegmatis* Δ(MsPmt) bacteria complemented with MmpS4[Cter]-PhoA (periplasmic control), MtPMT[Cter]-PhoA, KatG[Cter]-PhoA (cytoplasmic control), PhoA-[Nter]MtPMT or untagged MtPMT.

	MmpS4[Cter]-PhoA	MtPMT[Cter]-PhoA	KatG[Cter]-PhoA	PhoA-[Nter]MtPMT	MtPMT
Culture replicates	0.02020	0.00730	0.00160	0.00090	0.00190
	0.02950	0.02390	0.00640	0.00070	0.00140
	0.01650	0.01480	0.00460	0.00085	0.00190
Mean	0.02207	0.01533	0.00420	0.00082	0.00173
SD	0.006698	0.008313	0.002425	0.000104	0.000289

Table data for graph figure 3a : Replicate values of site-directed mutagenesis analysis of the amino acids D74, D176, R441 and Y444.

	Clone1 « culture 1 » replicate		Clone2 « culture 1 » replicate		Clone2 « culture 2 » replicate		Mean	SD
WT	95	106	115	85	108	91	100.00	11.4543
No PMT	-1.85	-2.17	-0.24	0.1	0.5	1.5	-0.36	1.4086
D74A	-1.53	1.05	0.73	3.21	0.1	-0.5	0.51	1.6112
D74N	7	4	0	-7	3	6	2.17	5.1153
D74E	3.31	2.34	6.86	7.18	8.5	7.6	5.97	2.5126
DE74-75ED	-0.97	-3.23	6.45	-1.29	-2.26	0.65	-0.11	3.4670
D176A	0.41	-0.56	2.99	0.65	0.5	0.68	0.78	1.1766
D176N	1.45	1.24	1.45	-1.45	-0.21	-1.86	0.10	1.5023
D176E	3.31	2.5	1.05	4.28	4.3	5.5	3.49	1.5676
R441A	0	3	3.23	2.26	0.65	-1	1.36	1.7274
R441L	-1	1	-0.83	1.24	0.83	1.86	0.52	1.1639
R441K	84	85.6	83.5	83	83.2	83	83.72	0.9968
Y444A	-1	0	-2	1	1.51	0.48	0.00	1.3046
Y444F	45	45.3	44.9	45	45.2	44.8	45.03	0.1862

Table data for graph figure 3b : Biological replicate values of the periplasmic phosphatase activity assessing the expression of the inactive MtPMT substituted mutant proteins fused to the PhoA tag .

	WT	D74N	D176N	R441L	Y444A
Culture replicates (%)	96	121	249,5	98,4	62,5
	104	118	251	138.4	74

Table data for graph figure 3d: *Biological replicate values of in-vivo* partial rescue of the activity of genetically inactivated enzyme R441A.

Treatment	None	2.5mM Imidazole	5mM Imidazole	2.5mM Guanidinium	5mM Guanidinium	
Culture Replicates (%)	99.47	105.07	103.2	99.73	107.47	WT
	100.27	95.2	101.33	106.67	103.73	
	0.27	12.27	27.73	20.27	29.07	R441A
	-0.27	11.2	15.73	18.4	21.6	

Table data for graph figure 5d : Replicate values of site-directed mutagenesis analysis of the amino acids within EL4^{Cter} domain.

WT	95	106	115	85	108	91
No PMT	-1	0	3	-3	1	0
W355A	28.07	35.75	31.56	23.18	35.06	38.55
W355F	7.12	2.93	-7.54	-4.05	0.84	-4.75
P359A	42	58.5	60	43	38	57
W362A	2	5	-13	1	5	4
W362F	17	8	10	8	14	14
M364A	82	82	84	78		
M364V	66.43	72.27				
S365A	133	140	122	138	104.95	108.91
S365T	101	100	97	103	94	106
L366A	79	89	83	88	81	88
Y371A	54	61	57	52	58.42	51.49
Y371F	105	105	91	89	82	104
I373Q	77.07	98.14	72.52	72.73	82.23	88.02
I373L	89.67	94.62	96.9	86.36	91.12	84.5
C381A	82	102	98			
C381S	88	84				
C386A	93.55	84.52	88.71	87.74	82.9	84.84
C386S	104	103	101	107		
C381-386A	94	87				
C381-386S	86	87	84	90		
L392A	34.7	26	31.8	23.5		
L392W	4	12	3.6	7	8.6	7.3
Y371A-L392A	2.3	2.4	5.6	1.6		
P396A	-2.65	3	0.14	3	6	5.5

Table data for graph figure 5e : Biological replicate values of the periplasmic phosphatase activity assessing the expression of the inactive MtPMT mutants of the EL4^{Cter} domain.

	REPLICATES (%)		
WT	100	96	104
W355A	148	152	144
W355F	220.6	88.2	
P359A	101.4	160.8	
W362A	57	63	
W362F	94.4	88.5	
Y371A	96	102	
Y371F	147	129	
L392A	86.3	99	
L392W	43.5	45	41
Y371A-L392A	72.5	74	70
P396A	67.7	40.6	
